# Förster resonance energy transfer-based kinase mutation phenotyping reveals an aberrant facilitation of Ca^2+^/calmodulin-dependent CaMKIIα activity in *de novo* mutations related to intellectual disability

**DOI:** 10.3389/fnmol.2022.970031

**Published:** 2022-09-01

**Authors:** Hajime Fujii, Hiroyuki Kidokoro, Yayoi Kondo, Masahiro Kawaguchi, Shin-ichiro Horigane, Jun Natsume, Sayaka Takemoto-Kimura, Haruhiko Bito

**Affiliations:** ^1^Department of Neurochemistry, Graduate School of Medicine, The University of Tokyo, Tokyo, Japan; ^2^Department of Pediatrics, Nagoya University Graduate School of Medicine, Nagoya, Japan; ^3^Department of Neuroscience I, Research Institute of Environmental Medicine (RIEM), Nagoya University, Nagoya, Japan; ^4^Department of Molecular/Cellular Neuroscience, Nagoya University Graduate School of Medicine, Nagoya, Japan; ^5^Department of Developmental Disability Medicine, Nagoya University Graduate School of Medicine, Nagoya, Japan

**Keywords:** CaMKII, intellectual disability, neurodevelopmental disorders, imaging, FRET, *de novo* mutation

## Abstract

CaMKIIα plays a fundamental role in learning and memory and is a key determinant of synaptic plasticity. Its kinase activity is regulated by the binding of Ca^2+^/CaM and by autophosphorylation that operates in an activity-dependent manner. Though many mutations in CAMK2A were linked to a variety of neurological disorders, the multiplicity of its functional substrates renders the systematic molecular phenotyping challenging. In this study, we report a new case of CAMK2A P212L, a recurrent mutation, in a patient with an intellectual disability. To quantify the effect of this mutation, we developed a FRET-based kinase phenotyping strategy and measured aberrance in Ca^2+^/CaM-dependent activation dynamics *in vitro* and in synaptically connected neurons. CaMKIIα P212L revealed a significantly facilitated Ca^2+^/CaM-dependent activation *in vitro*. Consistently, this mutant showed faster activation and more delayed inactivation in neurons. More prolonged kinase activation was also accompanied by a leftward shift in the CaMKIIα input frequency tuning curve. In keeping with this, molecular phenotyping of other reported CAMK2A *de novo* mutations linked to intellectual disability revealed aberrant facilitation of Ca^2+^/CaM-dependent activation of CaMKIIα in most cases. Finally, the pharmacological reversal of CAMK2A P212L phenotype in neurons was demonstrated using an FDA-approved NMDA receptor antagonist memantine, providing a basis for targeted therapeutics in CAMK2A-linked intellectual disability. Taken together, FRET-based kinase mutation phenotyping sheds light on the biological impact of CAMK2A mutations and provides a selective, sensitive, quantitative, and scalable strategy for gaining novel insights into the molecular etiology of intellectual disability.

## Introduction

Intellectual disabilities (ID) are prevalent in approximately 1% of the world population, and genetic as well as environmental factors play critical roles in ID pathogenesis (Vissers et al., 2016). Recently, *de novo* mutations in a key synaptic enzyme CAMK2A (Ca^2+^/calmodulin (CaM)- dependent protein kinase II alpha, CaMKIIα) have been shown to be associated with ID (Küry et al., 2017; Akita et al., 2018). CaMKIIα, which regulates synaptic plasticity, learning, and memory, is a protein kinase that is activated by Ca^2+^ transients caused by synaptic inputs or neuronal firing and modulates neuronal circuit properties *via* phosphorylation of key synaptic substrates leading to the up-regulation of AMPA-type glutamate receptor functions (Lisman et al., 1997, 2012; Woolfrey and Dell'Acqua, 2015; Takemoto-Kimura et al., 2017; Bayer and Schulman, 2019). Autoinhibitory domains self-inhibit their kinase activity under the baseline conditions, but are unblocked when Ca^2+^/CaM-binding is triggered, thus transforming neuronal activity into spatiotemporal domains of biochemical signaling (Bayer and Schulman, 2019). Upon autophosphorylation at threonine 286, an autonomous activity is achieved in which the kinase remains active even after the cessation of a Ca^2+^ rise (Hudmon and Schulman, 2002). Previously, it was determined that high-frequency neuronal stimuli facilitate this autonomy state in neurons (De Koninck and Schulman, 1998; Fujii et al., 2013). Frequency-tuning of CaMKIIα integrates synaptic inputs during rapid learning and is thought to underlie the multifaceted roles of CaMKIIα in learning and memory (Bayer and Schulman, 2019; Fujii and Bito, 2022).

Relatively small disturbances in CaMKIIα expression cause significant brain dysfunction. Consistently, previous biochemical studies examining threonine 286 autophosphorylation in various ID-related *de novo* CAMK2A mutations suggested that some mutants were indeed upregulated, while others were downregulated (Küry et al., 2017; Akita et al., 2018). However, whether these mutations affected the key molecular phenotype, namely Ca^2+^/CaM-dependent activation and frequency-tuning, of CaMKIIα have not been tested.

With a view to achieving a mechanistic understanding of ID and to begin to identify novel disease-modifying therapeutic strategies, in this study, we developed a quantitative molecular phenotyping pipeline of ID-related *de novo* CAMK2A mutations. First, we identified an ID patient with a *de novo* CAMK2A Pro212Leu (P212L) mutation. In keeping with three previously identified cases, our patient with P212L mutation had a mild clinical phenotype, showing moderate ID and autistic features, but no dysmorphic features and no seizure events. Previous *in vitro* studies, however, have failed to identify any dysregulation of CaMKIIα molecular phenotype as the heterologous expression of P212L mutant protein showed no change in protein expression or threonine 286 autophosphorylation levels (Küry et al., 2017).

To overcome this lack in molecular resolution, we built an analysis pipeline to quantitate the Ca^2+^/CaM-dependent activation of CaMKIIα and uncover possible alteration in its frequency-tuning in neurons. We developed a Förster resonance energy transfer (FRET)-based optical molecular phenotyping system, in which we combined an optical CaMKIIα FRET sensor hK2α with multiple wavelength optical interrogation devices to analyze Ca^2+^/CaM-dependent activation in CaMKIIα mutants related to ID.

## Materials and methods

The studies involving human participants were reviewed and approved by the Ethics Committee of the Nagoya University Graduate School of Medicine. Written informed consent to participate in this study was provided by the participants' parents. All recombinant DNA and animal experiments in this study were performed in accordance with the regulations and guidelines for the care and use of experimental animals at the University of Tokyo and approved by the institutional review committees of the University of Tokyo Graduate School of Medicine.

### Whole-exome sequencing

Genomic DNA was extracted from peripheral blood mononuclear cells using the QIAamp DNA Blood Mini Kit (Qiagen, Hilden, Germany). Trio-WES (patient, father, and mother) for the patient was performed using the Sure Select Human All Exon V6 kit for capture (Agilent Technologies, Santa Clara, CA, USA) and a HiSeq2500 system (Illumina, Inc., San Diego, CA, USA) for sequencing 101-bp paired-end reads. Obtained reads were aligned to the hg19 reference genome using the Burrows–Wheeler aligner (BWA, http://bio-bwa.sourceforge.net/) with default parameters and a –mem option. Polymerase chain reaction duplicates were removed using Picard tools (http://broadinstitute.github.io/picard/). Sequence variations were detected and annotated using VarScan2 (Koboldt et al., 2012) and ANNOVA R (Wang et al., 2010), respectively. For germline variations, we removed common single-nucleotide polymorphisms (SNPs) (defined as those with >1% allele frequency) using ExAC (http://exac.broadinstitute.org/), gnomAD (https://gnomad.broadinstitute.org/), 1,000 genomes (http://www.1000genomes.org/), ESP6500 (http://evs.gs.washington.edu/EVS/), and an in-house database. A conclusive assessment of molecular variants was performed according to guidelines issued by the American College of Medical Genetics and Genomics (ACMG) (Richards et al., 2015).

### Sanger sequencing of *de novo* mutation

After genome extraction, the fragment from *CAMK2A* (NG_047040.1), including the mutation site, was amplified using PrimeSTAR GXL DNA polymerase (Takara) with primers, 5'-GGTTTGCAGGGACTCCTG-3' (forward) and 5'- CTGGTCAGTCTTCATGCTC-3' (reverse). Sanger sequencing was performed using the PCR fragments with the same forward primer.

### Plasmid construction

The human *CAMK2A* clone (NM_171825.2) was purchased from GenScript. To construct humanized CaMKIIα FRET probe hK2α, a fragment containing kinase, regulatory and variable linker domain of CaMKIIα, mVenus, and a fragment containing variable domain to association domain of CaMKIIα were amplified by PCR using 5′-GGACTCAGATCTCGAGCCAGGATGGCCACCATCACCTGC-3′ and 5′-CTTCACACCATCGCTCTT-3′, 5′-AGCGATGGTGTGAAGGGTGGCGTGAGCAAGGGCGAGGAG-3′ and 5′-GCTCTCTGAGGATTCGCCACCCTTGTACAGCTCGTCCAT-3′, and 5′-GAATCCTCAGAGAGCACC-3′ and 5′-TAGATCCGGTGGATCCTCAGTGGGGCAGGACGGAGGG-3′, respectively. The three fragments were assembled between XhoI and BamHI sites of pmCerulean-C1 to give pN1-hK2α. The hK2α containing *de novo* mutations was generated by quick change cite-directed mutagenesis methods. Forward and reverse primers used for each mutants were F98S: 5′-GGTGGGGAACTGTCTGAAGATATCGTG-3′ and 5′-CACGATATCTTCAGACAGTTCCCCACC-3′; E109D: 5′-GAGTATTACAGTGACGCGGATGCCAGT-3′ and 5′-ACTGGCATCCGCGTCACTGTAATACTC−3′; A112V: 5′-AGTGAGGCGGATGTCAGTCACTGTATC-3′ and 5′-GATACAGTGACTGACATCCGCCTCACT-3′; E183V: 5′-TATCTCTCCCCAGTAGTGCTGCGGAAG-3′ and 5′-CTTCCGCAGCACTACTGGGGAGAGATA-3′; P212L: 5′-GTTGGGTACCCCCTGTTCTGGGATGAG-3′ and 5′-CTCATCCCAGAACAGGGGGTACCCAAC-3′; P212Q: 5′-GTTGGGTACCCCCAGTTCTGGGATGAG-3′ and 5′-CTCATCCCAGAACTGGGGGTACCCAAC−3′; P235L: 5′-GATTTCCCATCGCTGGAATGGGACACT-3′ and 5′-AGTGTCCCATTCCAGCGATGGGAAATC-3′; H282R: 5′-GCATCCTGCATGCGCAGACAGGAGACC-3′ and 5′-GGTCTCCTGTCTGCGCATGCAGGATGC−3′; T286P: 5′-CACAGACAGGAGCCCGTGGACTGCCTG−3′; and 5′-CAGGCAGTCCACGGGCTCCTGTCTGTG−3′. Sequences of PCR-amplified region were confirmed by Sanger sequencing service (FASMAC, Japan). For the expression of cultured neurons, hK2α was subcloned under the CaMKII promoter to obtain pCaMKII-hK2α.

### *In vitro* fluorescent plate reader

For FRET measurement in cell lysate, HEK293T cells (CRL11268, ATCC) were cultured in Dulbecco's Modified Eagle's Medium (D5796, Sigma-Aldrich) supplemented with fetal bovine serum and penicillin–streptomycin in 6-well-plates (IWAKI) and transfected with probe plasmids using XtremeGENE 9 (6365809001, Merck). Two to three days after transfection, cell lysates were collected with buffer containing 40 mM HEPES-Na, pH 8.0, 0.1 mM EGTA, 5 mM magnesium acetate, 0.01% Tween 20, sonicated using a sonicator (MICROSON ULTRASONIC CELL DISRUPTOR, XL2000-600, Misonix), and then centrifuged (TOMY, MX-300) to collect the supernatant. The protein concentration of the supernatant was determined by the Pierce BCA Protein Assay Kit (23227, Thermo Fisher Scientific) according to the manufacturer's protocol using the dilution series of pierce bovine serum albumin standard ampules, 2 mg/mL as the standard. The absorbance at 570 nm was quantified in a plate reader (IWAKI EZS-ABS Microplate Reader). To quantify the relative concentration of the probes in each sample, YFP fluorescence was measured by excitation at 510 nm and emission at 560 nm using a fluorescent plate reader (Infinite 200 PRO, Tecan).

*In vitro* FRET measurements were performed in 96-well-plates (PerkinElmer) and the probes were excited by 435 nm and measured by 485 nm for the CFP channel and were excited by 435 nm and measured by 535 nm for the YFP(FRET) channel, respectively. Lysate prepared from cells transfected with an empty vector was used for background subtraction. Total protein concentrations were adjusted to 70 or 80 μg/ml using the empty vector lysate, and the relative fluorescent intensities of each mutant sample were adjusted to give similar conditions between the mutants. As expression levels of E183V and P212Q were lower compared to other mutants, E183V, P212Q, and WT were compared in separate low expression groups, and other mutants (F98S, E109D, A112V, P212L, P235L, H282R, T286P) and WT were compared in the high expression group in **Figure 5**. For comparison of WT, P212L, and P212Q, the probe concentrations were adjusted to that of P212Q. Measurements were started at the volume of 99 μl including the final 0.03 to 3 μM of bovine calmodulin (Millipore or FUJIFILM Wako Chemicals) or a vehicle was added, and binding reactions were initiated by the addition of 2 μl of 0.15 mM CaCl_2_ (Nacalai Tesque, final 0.3 mM, 0.2 mM of free Ca^2+^ in the presence of 0.1 mM EGTA) and stopped by the addition of 2 μl of 0.25 mM EGTA (Nacalai Tesque). For Ca^2+^/CaM vs. response plots, the mean FRET ratio of the last three points before the addition of EGTA was plotted against added CaM concentration.

### Multiplex imaging of living neuron

Dissociated hippocampal neurons were prepared from the CA1/CA3 regions of the hippocampus of P0 Sprague–Dawley rat pups (Japan SLC) as described previously. At 9 days *in vitro*, neurons were co-transfected with plasmids encoding hK2α and R-CaMP2 under CaMKII promoter using Lipofectamine 2000 (Thermo Fisher Scientific). After 4–6 days, the neurons were subjected to live cell imaging in Mg^2+^-free Tyrode's solution (129 mM NaCl, 5 mM KCl, 30 mM glucose, 25 mM HEPES-NaOH, pH 7.4, 2 mM CaCl_2_; osmolality was adjusted to that of the conditioned culture medium using sucrose) supplemented with 0.5 mM MNI-glutamate (Tocris Bioscience) and 1 μM TTX (Tocris Bioscience) to prevent contamination from spontaneous and recurrent activity. For the measurement of memantine dose-response curves, 1, 10, and 100 μM memantine hydrochloride (Tokyo Chemical Industry) or vehicle (water) was added to the imaging solution. Neurons were maintained at around 37°C in a stage incubator (Tokai Hit) during all imaging sessions.

Neuronal cell bodies and dendritic spines were imaged using an inverted microscope (IX81, Olympus) equipped with an EM-CCD camera (C9100-13, Hamamatsu Photonics). UV photolysis of MNI-glutamate was performed with a 100x objective (UPlanSApo 100, NA 1.40, Olympus) and a 355-nm UV pulse laser (Polaris II, New Wave Research) that was controlled with a UV photolysis system (Hamamatsu Photonics) operated on an AQUACOSMOS software platform (Hamamatsu Photonics). Uncaging pulse rates were varied from 2.5 to 20 Hz with the use of a Master-8 pulse stimulator (A.M.P.I.). Excitation filters were FF01-438/24 (Semrock) for hK2α excitation and ET555/20 × (Chroma) for R-CaMP2 excitation, and each of the probes was sequentially excited with the use of filter exchanger OSP-EXA (Olympus) equipped with a mercury lamp (Olympus, USH-103OL). The emission filter was FF01-483/32 (Semrock) for the CFP channel and a combination of long-pass BA510IF (Olympus) and multiband band-pass fluorescence filter (FF01-433/517/613; Semrock) for YFP and R-CaMP2 channels. Camera exposure time was 10 ms for both hK2α and R-CaMP2, and the data acquisition rate was about 25 Hz for neuronal soma imaging. For dendritic spine measurements, the exposure time was 100 ms and the acquisition rate was about 4 Hz. For comparison of baseline CFP/YFP ratio in the neuronal soma, neurons expressing hK2α probes were live-imaged in normal Tyrode's solution (129 mM NaCl, 5 mM KCl, 30 mM glucose, 25 mM HEPES-NaOH, pH 7.4, 2 mM CaCl_2_, 1 mM MgCl_2_; osmolality was adjusted to that of the conditioned culture medium using sucrose) supplemented with 1 μM TTX (Tocris Bioscience) using 10x objective (UPlanApo 10x, NA 0.40, Olympus), FF01-438/24 (Semrock) for excitation, FF01-483/32 (Semrock) for CFP emission, and FF01-542/27 (Semrock) for YFP emission. Data acquisition and ROI analysis were performed blindly in genotypes of hK2α, except for memantine pharmacology experiments where about half of the data are acquired in open-label and the remaining half of the data were collected in blind in genotypes, drugs, and concentrations. Since the results from open- and blind-label experiments were similar, data from the open and blind labels were pooled and analyzed together in memantine pharmacology in **Figure 8**. For kinetics comparison in **Figure 3**, a neuron expressing WT hK2α that showed little R-CaMP2 signal was excluded from the analysis, and CFP fluorescence intensities during the baseline period measured under the same acquisition conditions were not significantly different between WT and P212L (8,163 ± 857 for WT, *n* = 19, 6913 ± 566 for P212L, *n* = 19, *p* = 0.23, unpaired *t-*test), suggesting the expression levels of the measured cells were similar. For comparison of 9 mutants and WT in **Figure 7**, a total of 16 neurons was measured in response to 5 and 20 Hz stimulations for each mutant in a blind manner, and R-CaMP2 responses were manually checked before the opening of the labels, and those cells that showed little R-CaMP2 responses at 20 Hz (one WT, one E183V, and two H282R cells), unstable baseline (one E183V cell), or experimental human error (one T286P cell) were excluded from the analysis. Quantitative analysis of images was performed using AQUACOSMOS (Hamamatsu photonics). ROI was made in the cell body, and the cell-free area of the acquired images and the CFP, YFP, and RFP fluorescence intensities were measured at each time point. After background subtraction, the data at the time of uncaging light was contaminated, which was detected by thresholding, removed, and interpolated by linear interpolation of the previous and following frames. The rolling average was performed on the average of 5 consecutive time points.

The FRET signal was calculated by taking the CFP/YFP ratio as R and the increment ΔR (=R-R_0_) from the pre-stimulus mean R_0_ was normalized by R_0_, giving a normalized FRET ratio ΔR/R_0_. The R-CaMP2 signal was normalized by the pre-stimulus mean fluorescence F_0_, and the difference ΔF from F_0_ was divided by F_0_, giving a normalized fluorescence change ΔF/F_0_. To compare kinetics, the maximum value of ΔR/R_0_ or ΔF/F_0_ during a period of about 20 s after stimulus onset was detected and defined as peak ΔR/R_0_ and peak ΔF/F_0_, respectively. To compare the activation kinetics, half rise time, which is the time to reach half the magnitude of peak amplitude, was calculated. Specifically, half rise time point was defined as the time from the stimulus onset to the midpoint between the first time point when the response trace exceeded half of the peak amplitude and the last time point when the response trace was under half of the peak amplitude, during the time period from stimulus onset until the peak amplitude was reached. Next, to compare the inactivation kinetics, half decay time, which is the time from peak to decay to half the peak amplitude, was calculated. Specifically, half decay time was defined as the time from the peak to the midpoint between the first time point when the response trace decayed to half of the peak amplitude and the last time point that the response trace was above half of the peak amplitude. ΔF/F_0_ images of hK2α and fluorescent images of R-CaMP2 in neurons were generated using Fiji. For neuronal soma, 10 average images were generated by excluding image frames containing artifacts due to uncaging, and ratiometric images were generated. The ratiometric images were normalized by dividing by the pre-stimulus R_0_ images. For localization analysis in dendritic spines, mean YFP fluorescence intensities of hK2α and mean R-CaMP2 fluorescence intensities during baseline periods before application of stimulations were used as proxies for the amount of hK2α and volume, respectively. Spine enrichment index was defined as (hK2α in spine / R-CaMP2 in spine) / (hK2α in adjacent shaft / R-CaMP2 in adjacent shaft). For hK2α and R-CaMP2 response measurements, those spines that showed a large decrease in FRET probe fluorescence (detected if CFP fluorescence was <85% of baseline or YFP fluorescence was less than 85% and CFP fluorescence was less than 90% of baseline after stimulation) or dim baseline fluorescence of R-CaMP2 (detected if baseline R-CaMP2 fluorescence intensities were less than twice of their standard deviations during the baseline period) were excluded from the analysis (WT had 17 decreased and 5 dim R-CaMP2 spines, P212L had 13 decreased and 2 dim R-CaMP2 spines excluded. Remaining 41 and 51 spines from 18 and 19 neurons were analyzed for WT and P212L, respectively). Peak amplitude was defined as the maximum of hK2α and R-CaMP2 during 5 frames from the end of stimulation. To create hK2α response images for 10 s before, immediately after, and 20 s after stimulation, CFP and YFP images were Gaussian-filtered [sigma (radius) = 1 in ImageJ], and CFP/YFP ratio images were created. It was then divided by the pre-stimulus average ratio image and thresholded with a cellular mask to create ΔR/R_0_ images. A total of 3 frames of ΔR/R_0_ images were averaged and displayed. Similarly, for R-CaMP2 images, 3 frames of ΔF/F_0_ images were averaged and displayed. Statistical analysis was performed using Excel (Microsoft), BellCurve for Excel (Social Survey Research Information Co., Ltd.), and EZR (Kanda, 2013) (Saitama Medical Center, Jichi Medical University, Saitama, Japan), a graphical user interface for R.

## Results

### Identification of *de novo* P212L mutation in CAMK2A from a patient with ID and ASD

An 18-month-old Japanese girl presented to our clinic because she was unable to walk independently. She was the first child born to healthy non-consanguineous parents. She was born at the gestational age of 40 weeks with a birth weight of 3,550 g (+1.42 SD) and a birth head circumference of 34.0 cm (+0.35 SD). The family history is unremarkable. She was able to sit after age 7 months, creep after 10 months of age, and was able to stand with support at 15 months of age. She was able to speak some meaningful words.

Initial physiological and neurological examinations at 18 months of age revealed no abnormalities except for mild hypotonia. No distinctive facial features were observed. Screening blood tests, including metabolic and karyotyping, were normal.

At age 2 years, she was able to walk independently, but her speech development was delayed. Her total score on the Modified Checklist for Autism in Toddlers (M-CHAT) was 3, indicating that she was at risk for autism spectrum disorder (ASD). Brain MRI at age 2 years was normal. At age 3 years, she was able to run and speak three-word sentences. At age 4 years, inattention behavior and difficulty in controlling affection were present. At age 5 years, her parents noted difficulty with reading and writing, including the inability to read numbers, and she was asking the same questions. She was diagnosed with moderate ID as well as ASD, attention-deficit/hyperactivity disorder (ADHD), and developmental coordination disorder, according to the Tanaka-Binet test (Tanaka, 1987) and clinical interview of the Diagnostic Interview for Social and Communication Disorders (DISCO) (Wing et al., 2002).

**Figure 1:**
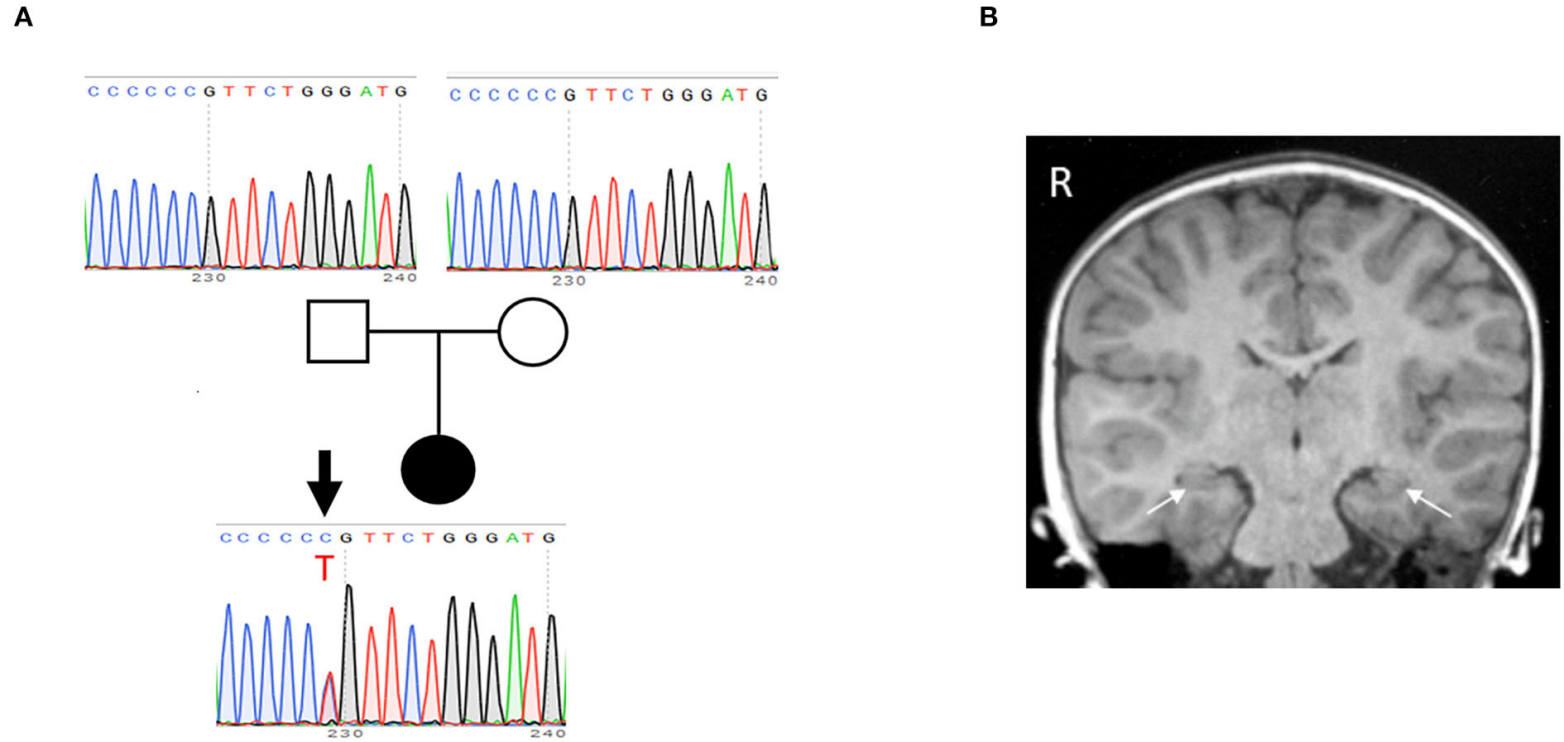
Identification of *de novo* P212L mutation in CAMK2A from a patient with ID and ASD. **(A)** The pedigree and electropherogram of the proband. Whole exome sequencing reveals a *de novo* heterozygous c.635C>T variant, which is confirmed by Sanger sequencing. **(B)** A coronal T1-weighted MR image at age 5 years shows no structural and volumetric abnormality, including hippocampi (arrows).

After obtaining written informed consent for genetic testing and publication of identifying information/images in an online open-access publication, trio-based whole exome sequencing identified a *de novo* heterozygous missense variant (NM_015981: c.635C>T; p.Pro212Leu) in *CAMK2A*, which was subsequently confirmed by Sanger sequencing (Figure 1A). This variant is not listed in public SNP databases, including ExAC (http://exac.broadinstitute.org/) and the Human Genetic Variation Database (http://www.hgvd.genome.med.kyoto-u.ac.jp/). The same variant is reported to be a pathogenic variant, which leads to an ID (Küry et al., 2017).

At age 5 years, a follow-up brain MRI showed no structural or volumetric abnormalities, including in the amygdalae and hippocampi (Figure 1B). Electroencephalography was normal. She had no other comorbidities such as epilepsy, infectious diseases, and visual and hearing impairments.

### Development of FRET-based optical molecular phenotyping system for mutational analysis of CAMK2A

**Figure 2:**
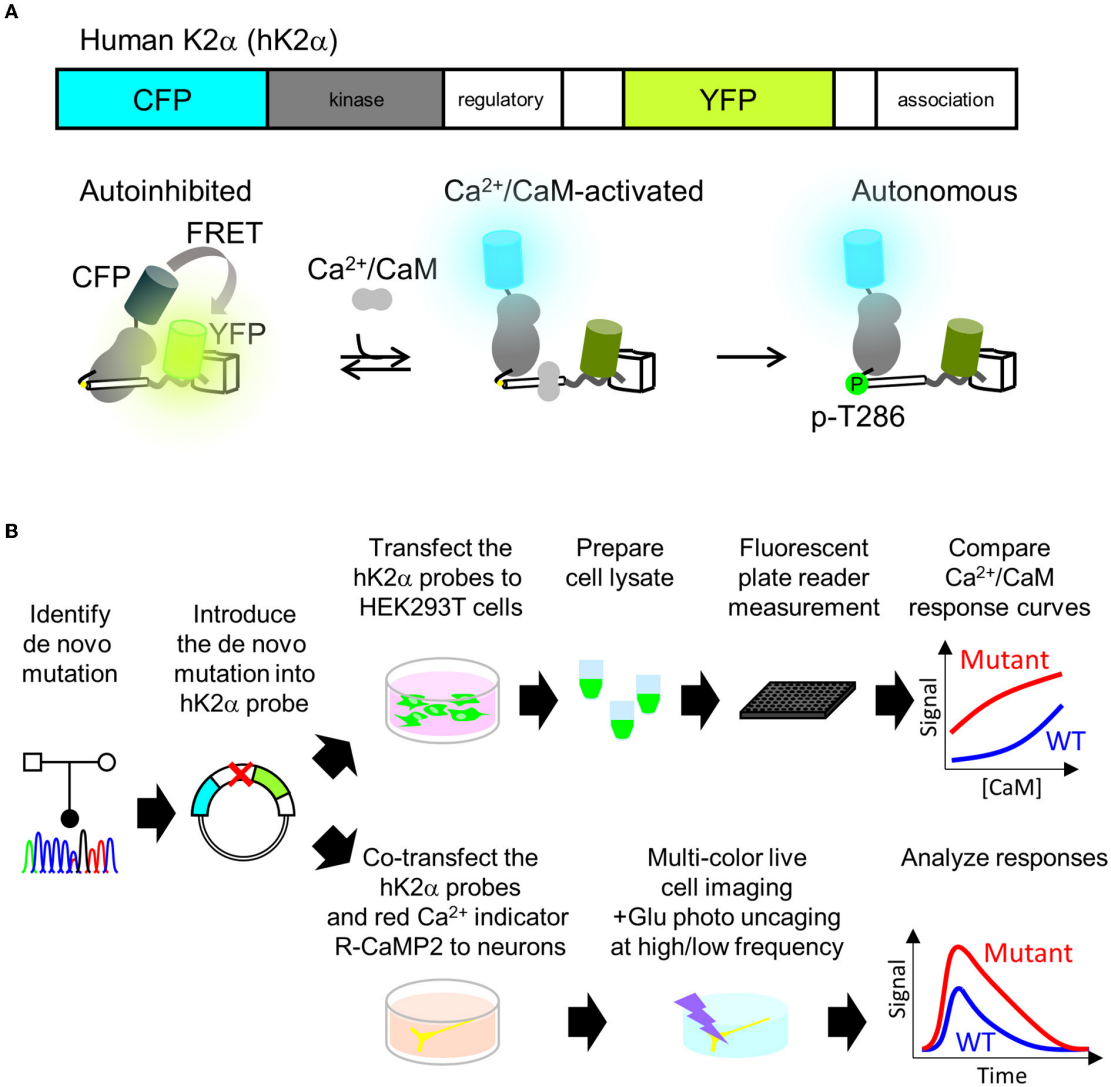
Plate reader FRET assay and multi-probe live neuron imaging analysis pipelines for analyzing *de novo* mutations in CAMK2A. **(A)** human CaMKIIα activation FRET probe hK2α. Schematics of primary structure (top) and expected conformational changes (bottom) of human CaMKIIα activation probe hK2α. Under baseline conditions, the kinase domain (gray) is autoinhibited by calmodulin-binding domain and autoinhibitory domain (white cylinder), and this conformation enhances FRET from CFP to YFP (bottom left, “autoinhibited”). Also, Ca^2+^/CaM binding relieves the autoinhibition, and thus the FRET from CFP to YFP is decreased (bottom middle, “Ca^2+^/CaM-activated). Subsequent autophosphorylation of threonine 286 keeps the activated conformation even after Ca^2+^/CaM dissociates from the kinase (bottom right, “autonomous”). Only one kinase subunit in the holoenzyme is illustrated for clarity. To show that activation of CaMKIIα is indicated by increase of the ratio, in this paper, CFP/YFP ratio R or ΔR/R_0_, normalized change from the baseline is presented. **(B)** Mutational analysis of CAMK2A gene for kinase activation profiles using plate reader FRET assay and multi-probe live neuron imaging. Identified mutations are introduced into hK2α FRET probe plasmid. For plate reader FRET assay, WT and mutant plasmids are transfected to HEK293T cells, lysates containing the probes are prepared and subjected to plate reader fluorescence measurements under various CaM concentrations to quantitatively compare the effect of the *de novo* mutations on Ca^2+^/CaM-dependent activation of CaMKIIα (top right). For a multi-probe live neuron imaging scheme, hK2α probes are co-transfected with red-color Ca^2+^ indicator R-CaMP2 into hippocampal dissociated cultured neurons. The transfected neurons are then subjected to multi-color live cell imaging under fluorescent microscopy to analyze responses against local glutamate uncaging delivered by various temporal patterns.

P212L mutation in a patient is the fourth report after three cases that have been reported (Küry et al., 2017), and thus it has been considered to be a recurrent *de novo* mutation in neurodevelopmental disorders. In addition, in the paralogous isoform CAMK2B, P213L mutation, which is in the same position as P212L in CAMK2A, has been found in a patient with neurodevelopmental disorders (Akita et al., 2018; Mutoh et al., 2022). These lines of clinical data strongly suggest the possibility that CAMK2A P212L mutation could alter the biochemical properties of CaMKIIα that underlie neurodevelopmental disorders. However, previous attempts had failed to reveal how P212L mutation affects biochemical properties of CaMKIIα, since protein expression levels and autophosphorylation level of threonine286 of P212L mutant under baseline conditions in heterologous cells or the migration of neuronal cells expressed with P212L mutant during cortical development were similar to those of WT (Küry et al., 2017). Although Ca^2+^/CaM-dependent activation is one of the most fundamental properties of CaMKIIα (Hudmon and Schulman, 2002; Lisman et al., 2012; Bayer and Schulman, 2019), previous studies had not examined how P212L mutation affects Ca^2+^/CaM-dependent activation, partly because of the lack of a sensitive, selective, quantitative, and scalable method to measure CaMKIIα activation. To meet these requirements, we took advantage of the humanized version of K2α, a FRET (Förster Resonance Energy Transfer)-based probe to monitor CaMKIIα activation that we developed previously (Fujii et al., 2013). Previously, CaMKIIα sensors that had one of the two fluorescent proteins attached to the C-terminus were developed (Takao et al., 2005; Lee et al., 2009), but to circumvent the potential folding problem (Shibata et al., 2015), K2α incorporated it in the middle of the flexible variable linker region exposed to the external surface of the holoenzyme (Fujii et al., 2013; Myers et al., 2017). Its FRET changes reported Ca^2+^/CaM-dependent activation as well as the autophosphorylation to threonine 286 (Figure 2A) (Fujii et al., 2013), which is another hallmark of CaMKIIα activation. Furthermore, its optical readout was shown to be quantitatively correlated with a conventional biochemical readout of CaMKIIα activation, and it was able to perform quantitative pharmacological analysis using the FRET signal, providing a rationale for the quantification (Fujii et al., 2013). Therefore, we analyzed the effect of mutation by introducing a mutation into the hK2α probe and comparing its signal change upon activation with WT hK2α. We took two schemes for the mutational analysis, (1) plate reader FRET assay, in which quantitative Ca^2+^/CaM-dependent activation curve of hK2α was obtained using *in vitro* high-throughput fluorescent plate reader and (2) multiplexed imaging of live neuron co-expressed with hK2α and red colorfast and linear Ca^2+^ indicator R-CaMP2 (Inoue et al., 2015) in response to various patterns of local glutamate uncaging stimulations (Figure 2B).

### P212L mutation aberrantly facilitated Ca^2+^/CaM-dependent CaMKIIα activation and rendered the intracellular CaMKIIα activation larger, faster and more sustained

**Figure 3:**
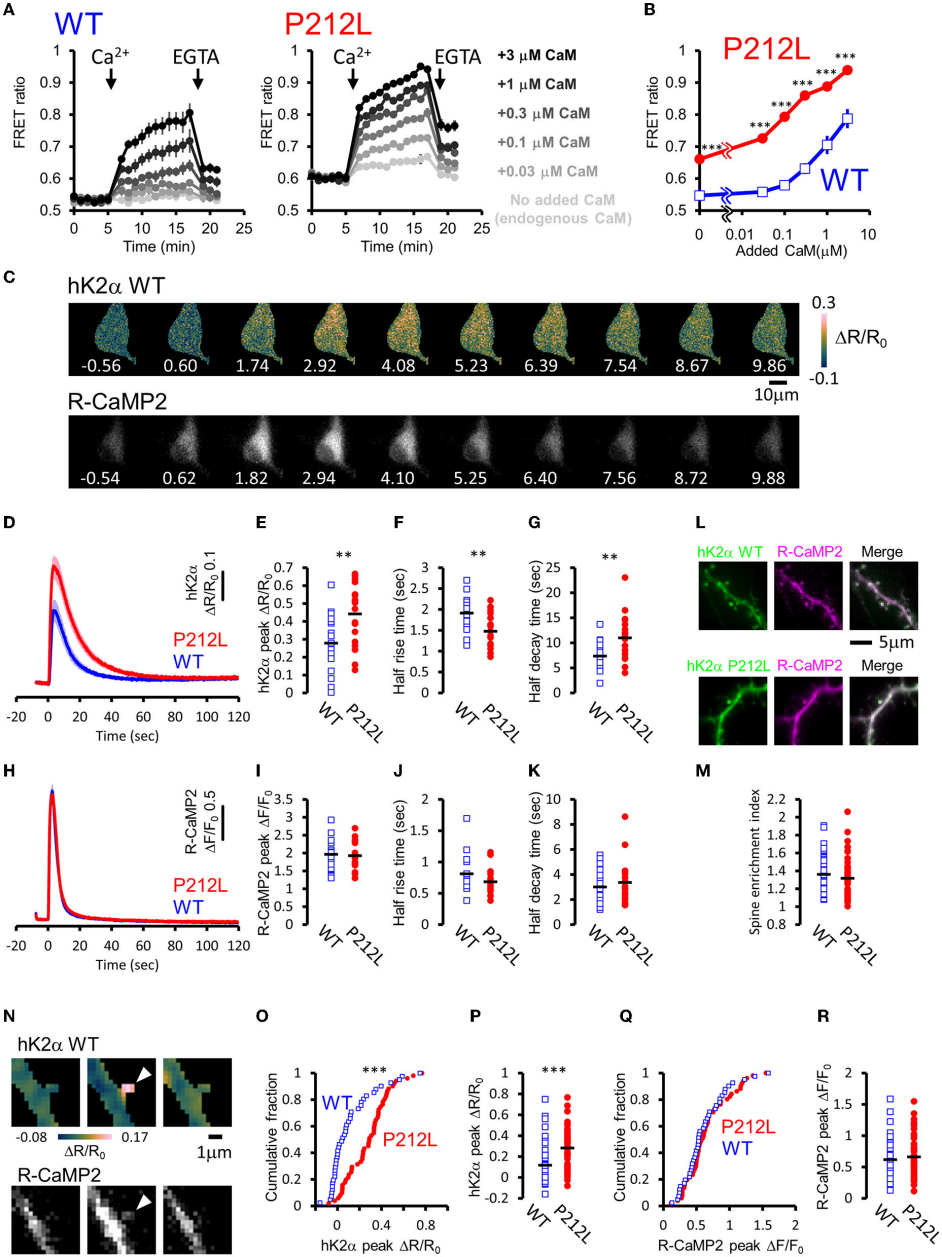
P212L mutation facilitated Ca^2+^/CaM-dependent activation of CaMKIIα, accelerates activation and decelerates inactivation processes. **(A)** Plate reader FRET measurement of WT (left) and P212L (right) mutant of hK2α under various concentrations of added CaM. Ca^2+^ and EGTA are added as indicated by arrows. Concentrations of added CaM are coded by the darkness of the traces, as indicated at the right. Mean ± s.e.m are shown (*n* = 4 for WT, *n* = 4 for P212L). **(B)** CaM concentration to FRET response plots for **(A)**. The mean of the last three points just before the addition of EGTA are plotted against CaM concentrations. Mean ± s.e.m are shown (*n* = 4 for each). *** *p* < 0.001 for WT vs P212L, at each CaM concentration, Tukey *post-hoc* analysis followed by ANOVA. **(C)** Representative images for multi-probe live neuron imaging. FRET ratio images of WT hK2α (top) and fluorescence images of R-CaMP2 (bottom) in response to local glutamate uncaging (50 photo-uncaging stimulations at 20 Hz) are shown. The time from the initiation of the stimulus sequence is indicated in seconds below in each image. **(D)** hK2α response traces of WT (blue) and P212L (red) against glutamate uncaging (50 stimulations at 20 Hz). Mean ± s.e.m are shown (*n* = 19 neurons for WT, *n* = 19 for P212L). **(E–G)** Comparison of hK2α response peak ΔR/R_0_ **(E)**, activation half rise time **(F)**, deactivation half decay time **(G)** between WT (blue open square) and P212L (red filled circle). Each plot represents data from each individual neurons, and black bar represents the mean. ** *p* < 0.01, unpaired *t*-test, *n* = 19 for WT *n* = 19 for P212L. **(H)** R-CaMP2 response traces in neurons co-transfected with hK2α WT (blue) and hK2α P212L (red) against glutamate uncaging (50 stimulations at 20 Hz). Mean ± s.e.m are shown by line and shaded areas, respectively (*n* = 19 neurons for WT, *n* = 19 for P212L). **(I–K)** Comparison of R-CaMP2 response peak amplitude **(I)**, activation half rise time **(J)**, deactivation half decay time **(K)** between neurons co-transfected with hK2α WT (blue open square) and hK2α P212L (red filled circle). Each plot represents data from each individual neuron, and the black bar represents the mean. n.s., not significant, unpaired *t*-test (*n* = 19 neurons for WT, *n* = 19 for P212L). **(L,M)** Comparison of spine enrichment of hK2α. **(L)** Representative images of hK2α (left, green), baseline R-CaMP2 (middle, magenta), and their merged images for WT (top) and P212L (bottom). **(M)** Comparison of spine enrichment (*p* = 0.323, unpaired *t*-test, *n* = 41 spines, 18 neurons for WT, *n* = 51 spines, 19 neurons for P212L). **(N–R)** Comparison of responses in dendritic spines. **(N)** Representative images for hK2α (top) and R-CaMP2 (bottom) responses ~10 seconds before the stimulation (left), immediately after the stimulation (middle), and ~20 seconds after the stimulation (right). **(O,P)** Cumulative distribution **(O)** and quantitative comparison **(P)** of hK2α responses between WT and P212L (*p* < 0.001, Kolmogorov-Smirnov test, *p* < 0.001, unpaired *t*-test, *n* = 41 spines from 18 neurons for WT, *n* = 51 spines from 19 neurons for P212L). **(Q,R)** Cumulative distribution **(Q)** and quantitative comparison **(R)** of R-CaMP2 responses between WT and P212L (*p* = 0.95, Kolmogorov-Smirnov test, *p* = 0.52, unpaired *t*-test, *n* = 41 spines from 18 neurons for WT, *n* = 51 spines from 19 neurons for P212L).

For plate reader FRET assay, cell lysates prepared from HEK293T cells expressing hK2α were mixed with CaM, and after measurement at baseline, Ca^2+^ was added to induce Ca^2+^/CaM-dependent activation. Both WT and P212L showed an increase in the CFP/YFP ratio (FRET ratio) upon the addition of Ca^2+^ (Figure 3A). Subsequent addition of EGTA lowered the increased FRET ratio (Figure 3A), confirming that FRET change represents Ca^2+^/CaM-dependent activation as in the previous study (Fujii et al., 2013). By changing the concentration of added CaM, CaM dose-response curves were obtained for WT and P212L (Figures 2A,B). P212L clearly showed enhanced Ca^2+^/CaM-dependent activation compared to WT under all the CaM concentrations examined (0, 0.03, 0.1, 0.3, 1, 3 μM). Furthermore, P212L clearly showed activation under 0.03 μM CaM, whereas WT activation occurred at 0.1 μM or higher CaM concentrations, indicating facilitated Ca^2+^/CaM-dependent activation in P212L (Figures 3A,B).

In neurons, CaMKIIα is activated by intracellular Ca^2+^ rises through NMDAR triggered by glutamate release, and it plays important roles in synaptic plasticity, learning, and neural development (Lisman et al., 2012), and the precise regulation of CaMKIIα activity is required for normal brain functions (Fujii and Bito, 2022). As hK2α P212L was activated at low concentrations of Ca^2+^/CaM, glutamate-input-dependent CaMKIIα activation in neurons may be enhanced by P212L mutation. To investigate this possibility, hK2α probes were transfected into hippocampal dissociated cultured neurons along with red Ca^2+^ indicator R-CaMP2 to check for Ca^2+^ rise induced by stimulation (Inoue et al., 2015). The cell bodies were stimulated by local glutamate uncaging in Mg^2+^-free solutions in the presence of TTX, which has been used to efficiently trigger NMDAR-mediated Ca^2+^ influx and CaMKII activation (Matsuzaki et al., 2004; Lee et al., 2009; Fujii et al., 2013), as we previously demonstrated that frequency-dependency of CaMKIIα was observed in soma as well as in dendritic spines (Fujii et al., 2013). High-frequency stimulation (50 uncaging stimuli at 20 Hz), which efficiently triggers CaMKIIα activation, resulted in a fast increase in the R-CaMP2 signal, followed by a slower increase in the hK2α FRET signal (Figure 3C). The hK2α signal persisted even after the R-CaMP2 signal returned to the baseline (Figures 3C,D,H), suggesting that CaMKIIα keeps activated conformation by autonomous state and CaM trapping, consistent with the previous studies (Meyer et al., 1992; Hudmon and Schulman, 2002; Lisman et al., 2012; Fujii et al., 2013; Bayer and Schulman, 2019). Strikingly, P212L showed about ~60% larger peak amplitude of hK2α compared to WT (ΔR/R_0_ of hK2α: 0.28 ± 0.039 for WT, 0.44 ± 0.038 for P212L, *p* < 0.01, unpaired *t*-test) (Figure 3E). Furthermore, hK2α activation was faster and more sustained in P212L compared to WT (Figure 3D). To quantitatively compare the kinetics, from the traces of each neuron, we calculated half-rise time, time from the start of stimulation to rise to half of the maximum amplitude, and the half-decay time, time from the peak to decay down to half of the maximum amplitude. These kinetics analyses demonstrated that P212L was activated about ~30% faster (half rise time: 1.9 ± 0.09 s for WT and 1.5 ± 0.09 s for P212L, p<0.01, unpaired *t-*test) and sustained the activation about ~50% longer time (half decay time: 7.4 ± 0.64 s for WT and 11.0 ± 1.02 s for P212L, *p* < 0.01, unpaired *t-*test) (Figures 3F,G). There were no significant differences in peak amplitude, half rise time, and half decay time of R-CaMP2 signals (ΔF/F_0_ of R-CaMP2: 2.0 ± 0.11 for WT, 1.9 ± 0.09 for P212L, *p* = 0.77, unpaired *t*-test; half rise time: 0.81 ± 0.08 s for WT and 0.68 ± 0.05 s for P212L, *p* = 0.13, unpaired *t*-test; half decay time: 3.0 ± 0.4 s for WT and 3.4 ± 0.4 s for P212L, *p* = 0.48, unpaired *t-*test) (Figures 3H–K). CFP/YFP baseline ratio were not significantly different between WT and P212L (0.55 ± 0.012 for WT, 0.56 ± 0.012 for P212L, *p* = 0.696, unpaired *t*-test), suggesting comparable baseline activation levels. Thus, our multi-probe imaging and quantitative analysis revealed that P212L mutation resulted in greater, faster, and more sustained CaMKIIα activation in the neurons.

Since biochemical reactions in the dendritic spine can be different from that of neuronal soma, considering the distribution of the molecules and small volumes (Kennedy et al., 2005), we compared hK2α probe localization and response in the dendritic spine. To examine localization, we compared the relative enrichment of the hK2α probe in the dendritic spine to the adjacent shaft using baseline fluorescence intensity R-CaMP2 before photo-stimulation as a proxy for volume marker (Figure 3L). hK2α showed enrichment in the dendritic spine relative to the dendritic shaft, consistent with the previous reports (Otmakhov et al., 2004; Zhang et al., 2008), but there was no significant difference between WT and P212L (1.36 ± 0.032 for WT, 1.32 ± 0.031 for P212L, *p* = 0.323, unpaired *t*-test) (Figure 3M). Next, hK2α and R-CaMP2 responses were measured against high-frequency uncaging stimuli (100 UV-uncaging stimuli delivered at 20 Hz) (Fujii et al., 2013). Consistent with the measurements in the neuronal soma, we observed that P212L showed larger responses in the stimulated dendritic spines (ΔR/R_0_ of hK2α: 0.12 ± 0.032 for WT, 0.28 ± 0.026 for P212L, *p* < 0.001, Kolmogorov-Smirnov test, *p* < 0.001, unpaired *t*-test) (Figures 3O,P), while R-CaMP2 showed no significant difference (ΔF/F_0_ of R-CaMP2: 0.62 ± 0.05 for WT, 0.66 ± 0.046 for P212L, *p* = 0.95, Kolmogorov-Smirnov test, *p* = 0.52, unpaired *t*-test) (Figures 3Q,R). Together, our results clearly demonstrated that a P212L mutation caused a facilitated CaMK2α activity, both in the soma and in the dendritic spines.

### Input frequency decoding properties of CaMKIIα are tuned to lower frequencies in P212L mutant

**Figure 4:**
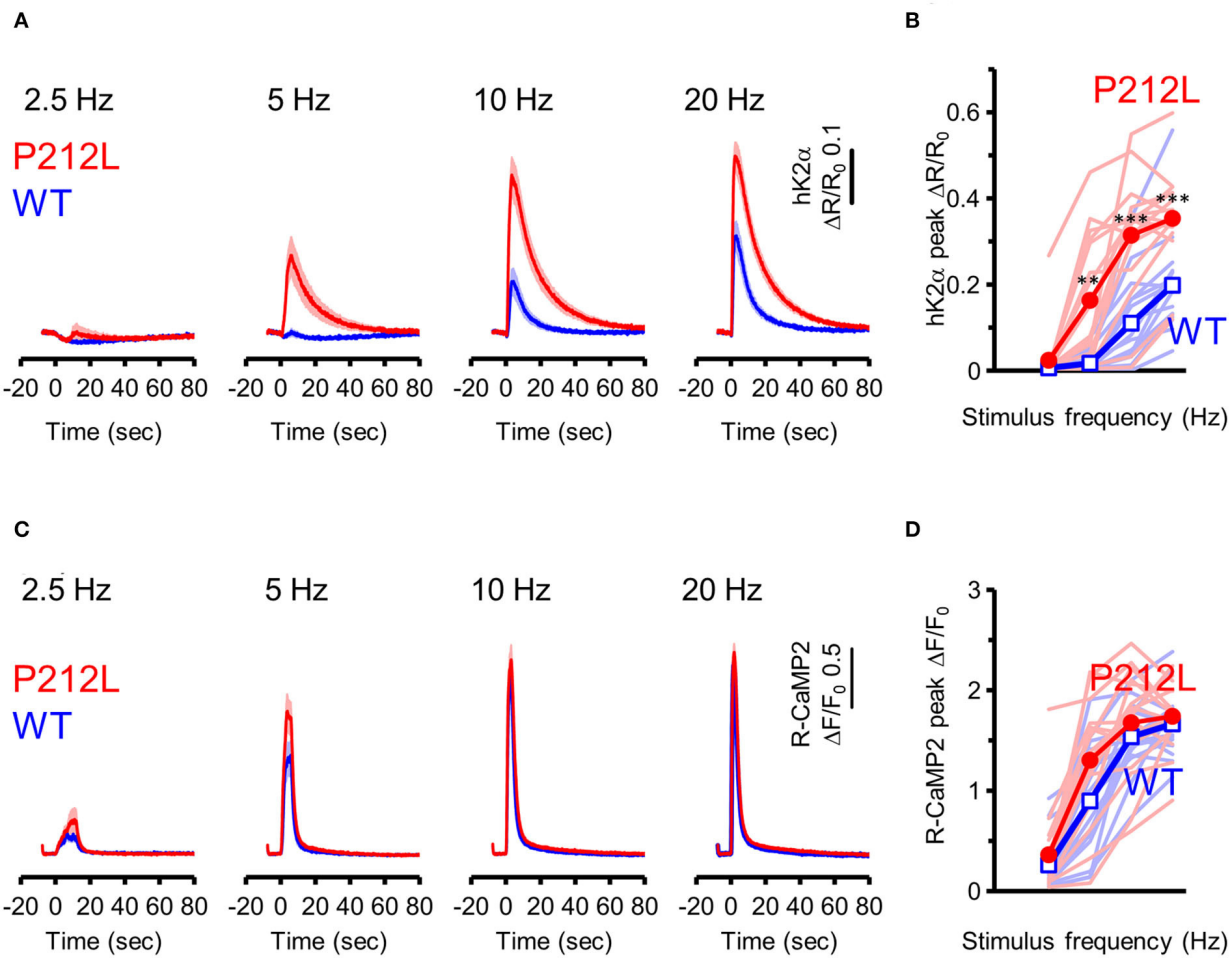
P212L mutation shifted frequency-dependent CaMKIIα activation curve toward lower stimulation frequencies. **(A)** hK2α WT (blue) or P212L (red) response traces in response to glutamate uncaging (30 stimulations at 2.5, 5, 10, 20 Hz). Mean ± s.e.m are shown, *n* = 16 for WT, *n* = 16 for P212L. **(B)** Comparison of hK2α response peak amplitude between WT (blue open square) and P212L (red filled circle). Each shaded line represents data from each individual neuron. **p < 0.01, *** *p* < 0.001, WT vs. P212L, repeated measures ANOVA followed by Tukey's test, *n* = 16 for WT, *n* = 16 for P212L. **(C)** R-CaMP2 responses to 30 stimulations delivered at 2.5, 5, 10, 20 Hz in neurons co-expressed with K2α WT (blue) or P212L (red). Mean ± s.e.m are shown (*n* = 16 for WT, *n* = 16 for P212L). **(D)** Comparison of R-CaMP2 response peak amplitude between WT (blue open square) and P212L (red filled circle). Each shaded line represents data from each individual neuron. *n* = 16 for WT, *n* = 16 for P212L.

Does the introduction of P212L only affect response amplitude and rise the decay kinetics of CaMKIIα activation? Or is it possible that there is some effect on the information processing properties of neural inputs? It has been biochemically demonstrated that CaMKIIα is activated depending on the frequency of Ca^2+^ pulses (De Koninck and Schulman, 1998) and accordingly, on the frequency of glutamate uncaging stimulation (Fujii et al., 2013). Such stimulus frequency-dependent activation of CaMKIIα may play an important role in the regulation of the induction of input frequency-dependent plasticity as well as learning and memory (Bach et al., 1995; Mayford et al., 1995; Rotenberg et al., 1996; Chang et al., 2017; Bayer and Schulman, 2019; Fujii and Bito, 2022). Since CaMKIIα activity sums up if the next input comes before the CaMKIIα activation returns to the baseline (Hanson et al., 1994; Chang et al., 2017; Bayer and Schulman, 2019), slower deactivation kinetics in P212L could possibly alter input frequency tuning of CaMKIIα. To investigate this possibility, glutamate uncaging stimulation was repeated 30 times at various frequencies (2.5, 5, 10, and 20 Hz), and multiplexed imaging of hK2α and R-CaMP2 was performed (Figures 4A–D). Consistent with the previous studies (Fujii et al., 2013), WT showed little response up to 5 Hz, but the response increased at 10 and 20 Hz, and a frequency-dependent activation response was observed (Figures 4A,B). In contrast, P212L showed lowered frequency tuning (Figures 4A,B). Although little response was observed at 2.5 Hz, 5 Hz stimulus triggered a more pronounced response compared to WT (Figures 4A,B). hK2α P212L reached nearly plateau level activation at 10 Hz (Figures 4A,B) (hK2α amplitude for WT and P212L: 2.5 Hz: 0.0061 ± 0.00082, 0.024 ± 0.016, *p* = 0.63; 5 Hz: 0.017 ± 0.0055, 0.16 ± 0.037, *p* < 0.001; 10 Hz 0.11 ± 0.025, 0.32 ± 0.036, *p* < 0.001; 20 Hz: 0.20 ± 0.029, 0.35 ± 0.028, *p* < 0.001; repeated measures ANOVA and Turkey's test, *n* = 16 for both WT and P212L). For R-CaMP2, significant dependency on the input frequency was observed in R-CaMP2 amplitude, but there was no difference between genotypes (repeated measures ANOVA, *p* < 0.001 for frequency, *p* = 0.1411 for genotype, *p* = 0.1419 for interaction). These results demonstrate that P212L mutation shifted the input tuning curve to a lower frequency and disrupted the information processing properties of the neurons.

### Aberrantly facilitated Ca^2+^/CaM-dependent activation is a prevalent molecular phenotype among CAMK2A mutants related to ID

Is the facilitated Ca^2+^/CaM-dependent activation a molecular phenotype of CaMKIIα solely observed in P212L? Or is it a phenotype that is also prevalent in other CAMK2A mutations found in ID? To answer this question, we took advantage of the high-throughput capability of our analytical pipeline and investigated a series of CAMK2A de novo mutations (F98S, E109D, A112V, E183V, P212L, P212Q, P235L, H282R, and T286P) identified from patients with ID (Küry et al., 2017; Akita et al., 2018).

In this analysis, to adjust for total protein concentration and probe concentration between the mutants being compared, 2 mutants (E183V and P212Q) that were particularly low in expression levels were compared to WT in one group, and 7 mutants (F98S, E109D, A112V, P212L, P235L, H282R, T286P) were analyzed in another group, although the WT response was almost identical between these conditions.

**Figure 5:**
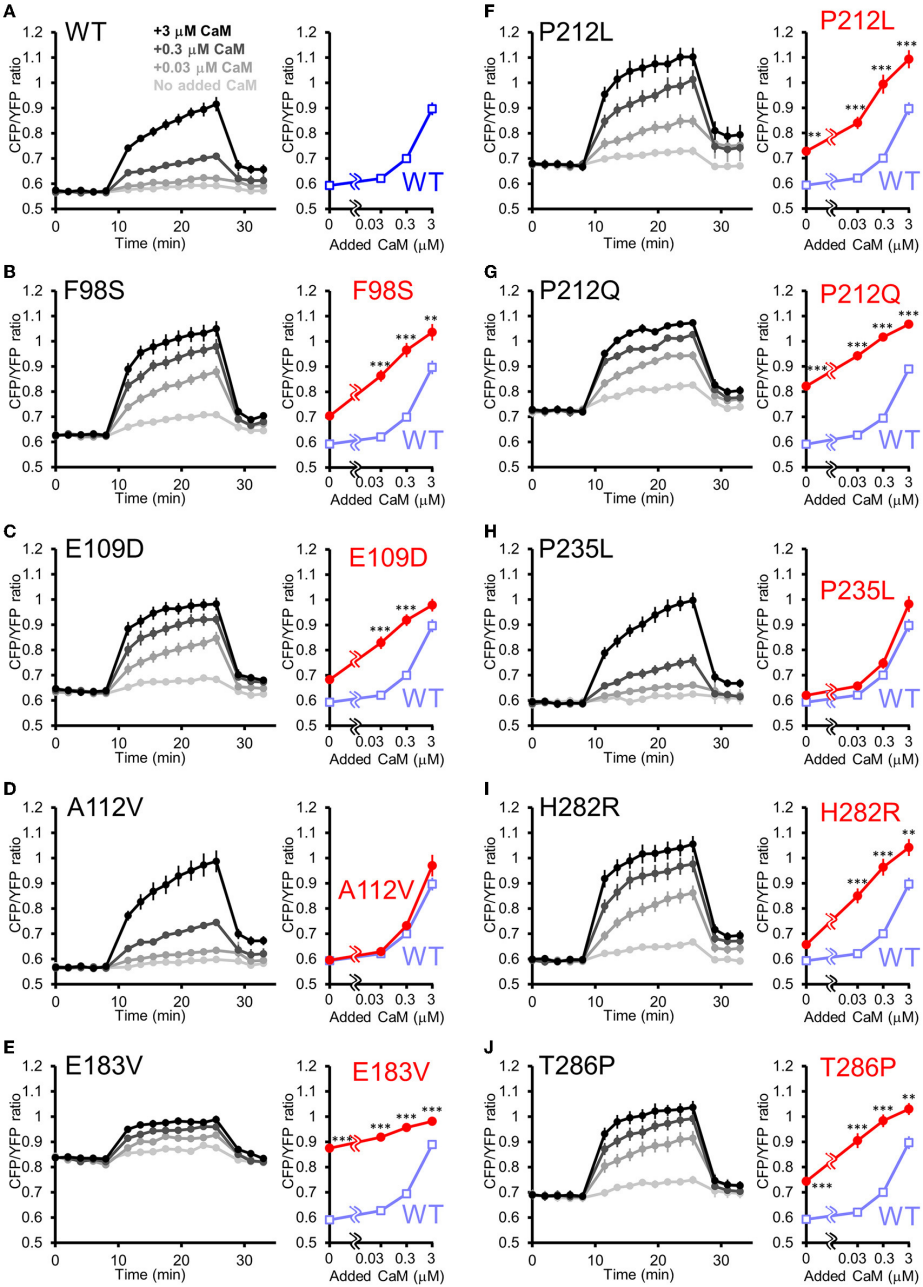
FRET plate reader analysis of effects of *de novo* mutations identified from ID on Ca^2+^/CaM-dependent activation of CaMKIIα. **(A–J)** Plate reader FRET assay (left) and CaM dose response curves (right) for hK2α WT and *de novo* mutants stimulated by 0, 0.03, 0.3, 3 μM added CaM. To ease comparison, dose-response curves of WT (shaded blue open square) are overlayed that of each mutant [red filled circle, **(B–J)**]. Mean ± s.e.m. are indicated, two-way ANOVA followed by *post-hoc* Turkey's test **p < 0.01, ***p < 0.001. *n* = 9 for WT, F98S, E109D, A112V, P212L, P235L, H282R and T286P and *n* = 14 for WT, E183V and P212Q.

The results revealed three qualitatively different types. First, 6 mutants (F98S, E109D, P212L, P212Q, H282R, and T286P) showed a CaM dose-response curve similar to that of P212L and were more activated than WT, especially at low Ca^2+^/CaM concentrations (Figures 5A–C,F,G,I,J). Next, 2 mutants (A112V, P235L) showed Ca^2+^/CaM dose-response curves similar to WT (Figures 5A,D,H). The remaining one mutant (E183V) showed an elevated CFP/YFP ratio even in the basal state, and the modulation by Ca^2+^/CaM concentration was small (Figure 5E). These data demonstrate that facilitated CaM-dependent activation was not P212L-specific but observed in 6 of the 9 CAMK2A *de novo* mutations related to ID.

**Figure 6:**
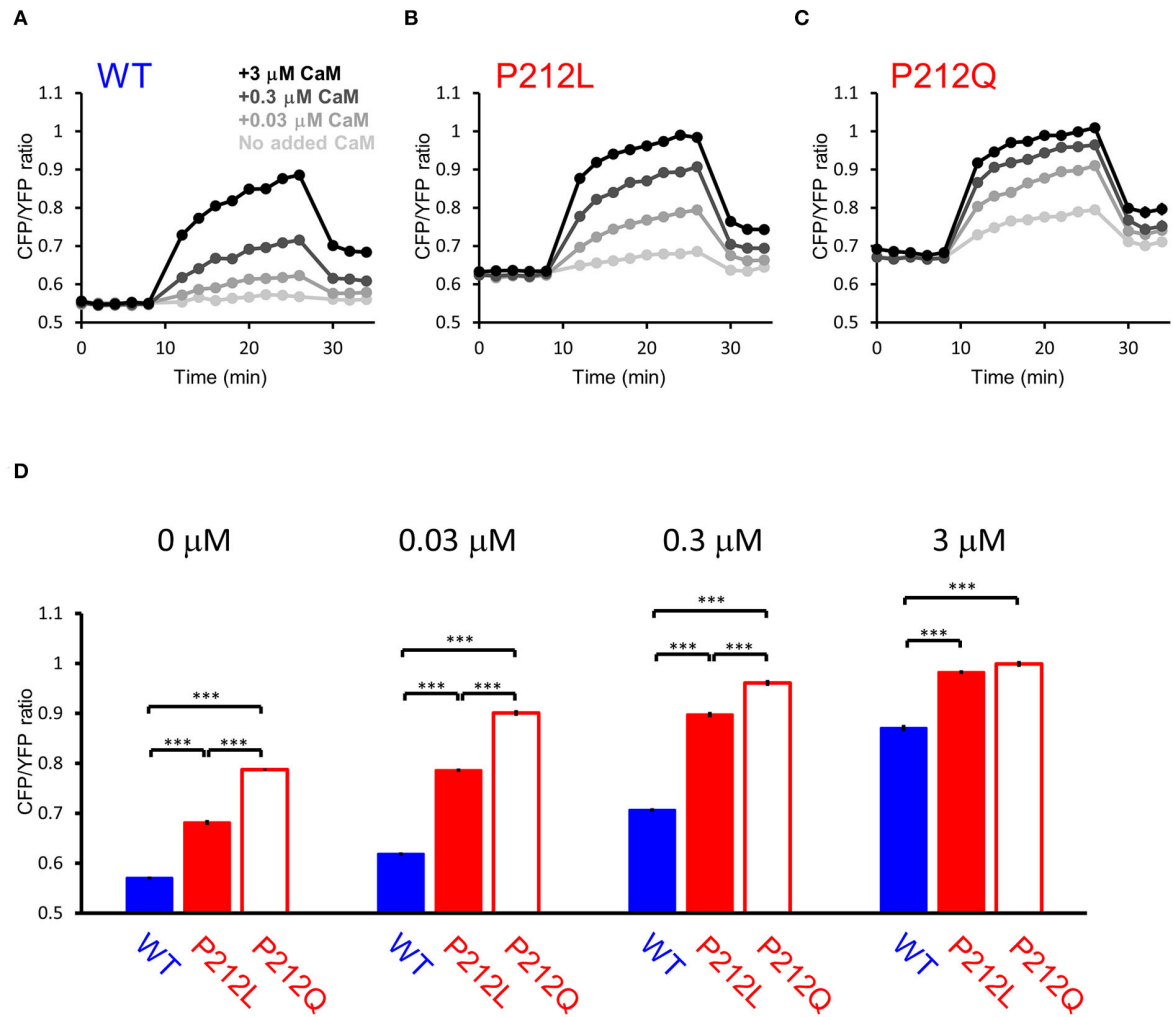
FRET plate reader analysis revealed quantitative differences in Ca^2+^/CaM-dependent activation between WT, P212L and P212Q. Plate reader FRET assay for WT **(A)**, P212L **(B)**, P212Q **(C)**, and their CaM dose responses **(D)** under 0, 0.03, 0.3, 3 μM added CaM. Mean ± s.e.m., two-way ANOVA followed by Turkey's test. ***p < 0.001. *n* = 18 for each plot, except for P212L under 3 μM CaM (*n* = 17), in which one measurement showed no response, which was considered to be a procedural error of Ca^2+^ addition and excluded.

Among the mutations analyzed, P212Q is a missense mutation that is altered at the same amino acid position as P212L. Although a small sample size precludes precise comparison, on comparing the current case with the reported case, the pathological phenotype of the patient with P212L reported here was milder compared to that of P212Q reported previously in terms of ID and the absence of seizures (Akita et al., 2018). Furthermore, autophosphorylation of threonine 286 was upregulated in P212Q, while P212L showed no significant difference (Küry et al., 2017; Akita et al., 2018). So, it can be predicted that facilitated Ca^2+^/CaM-dependent activation is more profound in P212Q compared to P212L. To elucidate if there is a quantitative difference between P212L and P212Q, we directly compared P212L, P212Q, and WT by FRET plate reader assay. The results showed that P212Q was more readily activated than P212L at low concentrations of CaM (Figure 6).

**Figure 7:**
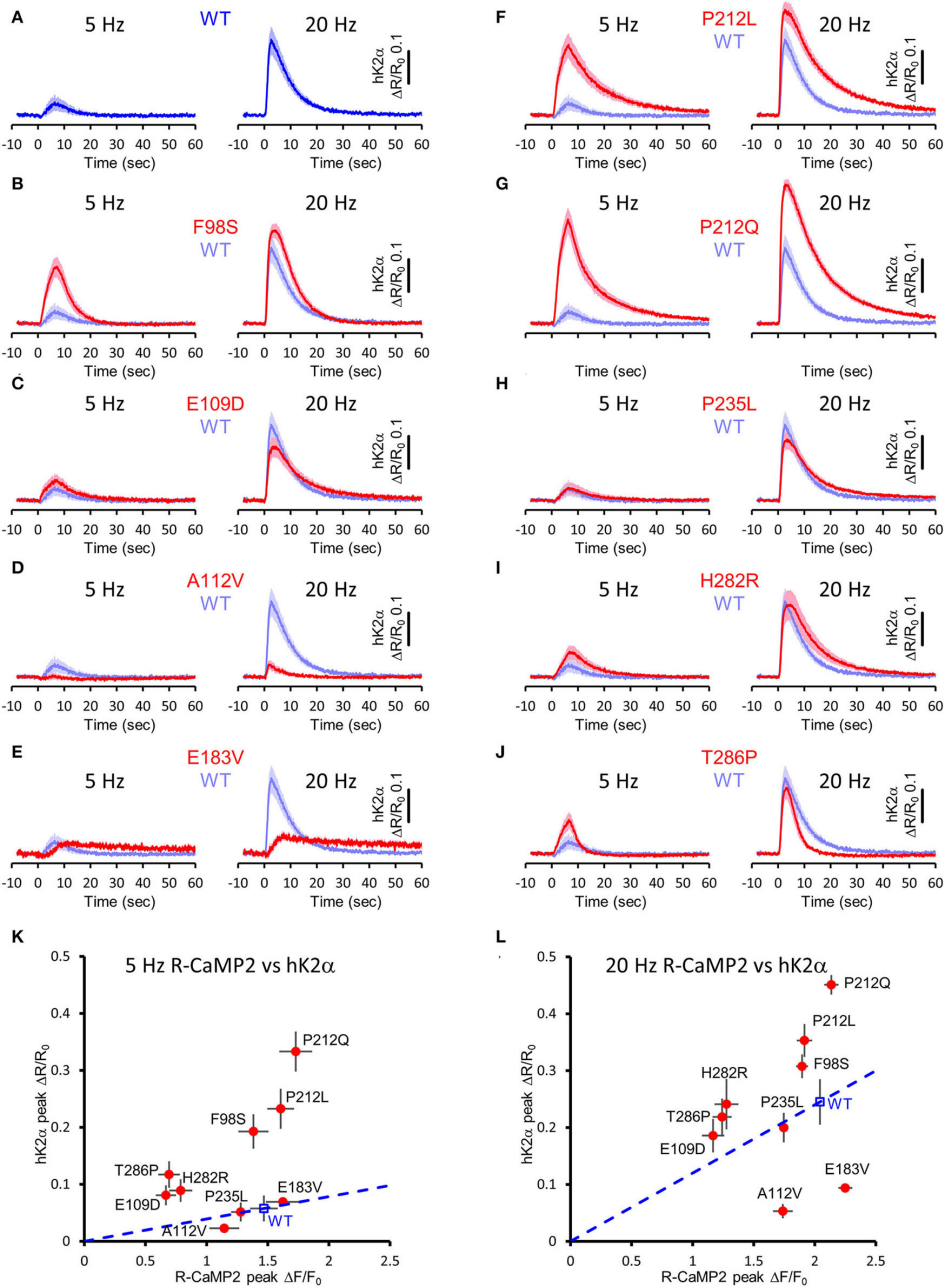
Activation profiles of CAMK2A *de novo* mutations associated with ID in living neurons. **(A–J)** hK2α activation traces in response to 30 photo-stimulations delivered at 5 Hz (left) or 20 Hz (right) To aid comparison, WT response traces (shaded blue traces) are overlayed in each mutant traces [red traces, **(B–J)**]. Mean ± s.e.m. are shown. *n* = 14 for E183V and H282R, *n* = 15 for WT, E109D, and T286P, *n* = 16 for F98S, A112V, P212L, P212Q, and P235L. **(K,L)** R-CaMP2 peak amplitude vs. hK2α peak amplitude plots. Mean ± s.e.m. are shown.

Next, to elucidate the activation profiles of the *de novo* mutants of CaMKIIα in living neurons, we performed multiplex imaging for a series of hK2α mutants and measured their responses to 30 glutamate uncaging stimulations delivered at 5 or 20 Hz (Figures 7A–J, Supplementary Figures 1, 2).

Under these conditions, R-CaMP2 peak amplitudes for E109D, H282R, and T286P were significantly lower than WT for both 5- and 20-Hz stimuli (Supplementary Figures 1, 2). A112V and P235L showed a slight but significant decrease in R-CaMP2 response against 20-Hz stimuli compared with WT (Supplementary Figure 2D). To best correct for the different Ca^2+^ levels between mutants, we took advantage of the fact that R-CaMP2 responds relatively linearly under a wide range of Ca^2+^ concentration (Inoue et al., 2015) and plotted hK2α peak amplitude against R-CaMP2 peak amplitude (Figures 7K,L). By considering a line connecting the WT plot and the origin (baseline conditions), the responses of mutants plotted above this line are considered to be enhanced, while the responses of mutants plotted below it are considered to be attenuated (Figures 7K,L, blue dash lines). Consistent with the results from FRET plate reader assays, F98S, E109D, P212L, P212Q, H282R, and T286P were plotted above these lines, P235L was almost overlayed with these lines, and E183V was plotted below these lines. A112V was unexpectedly plotted under the line, and the response was significantly lower that of WT under 20 Hz stimulation, suggesting that processes other than Ca^2+^/CaM-dependent activation could be impaired in neuronal conditions. Taken together, aberrantly facilitated Ca^2+^/CaM-dependent CaMKIIα activation was observed in two-thirds of ID-related *de novo* CAMK2A mutations reported so far, and is considered to be the prevalent molecular phenotype in ID.

### Attenuation of NMDAR signaling normalized aberrant activation of P212L

**Figure 8:**
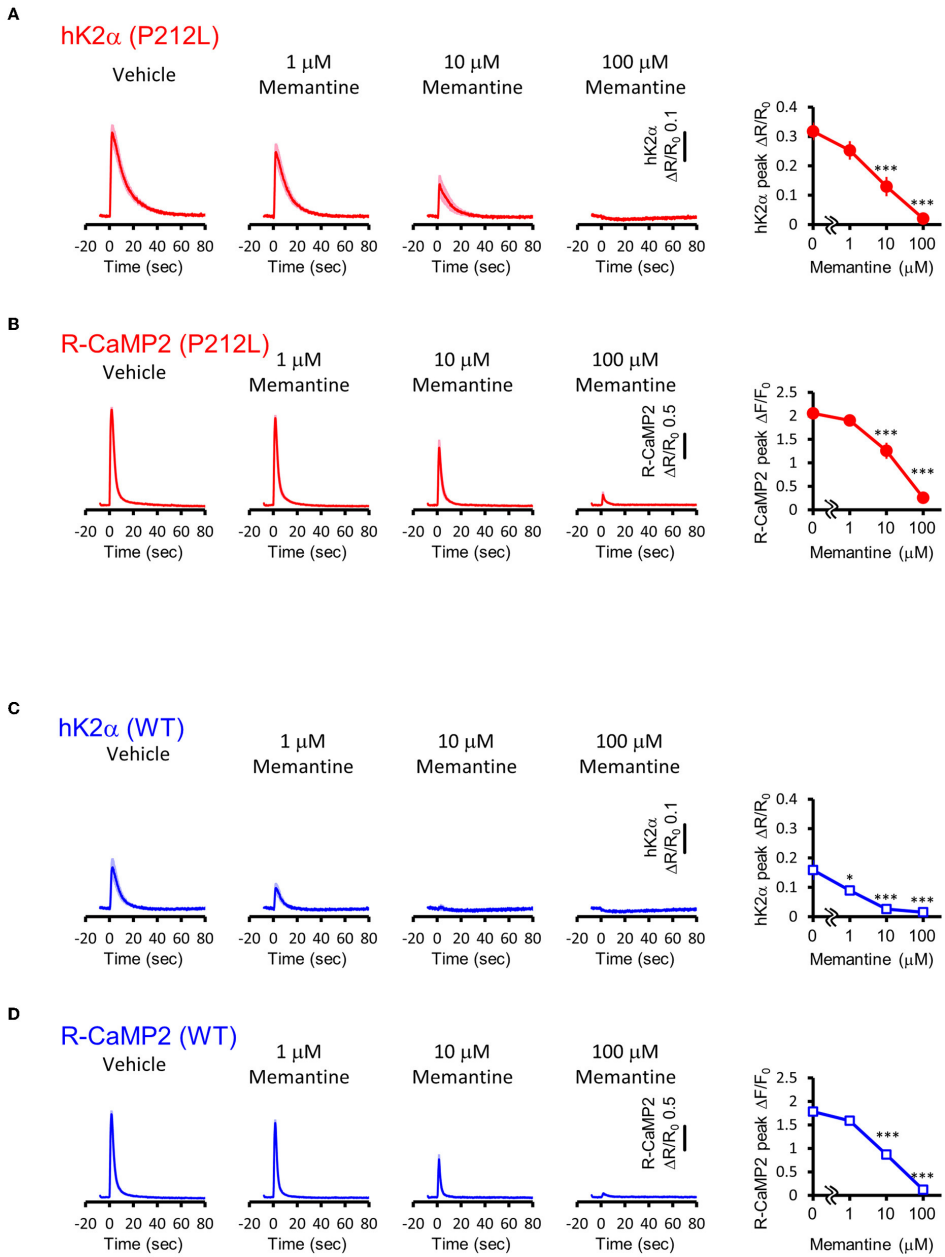
Memantine suppressed augmented activation of P212L. **(A)** Activation traces (left) and dose-response curve (right) of hK2α P212L in response to 30 photo-stimulations delivered at 20 Hz under 0, 1, 10, 100 μM memantine. *n* = 22 for 0 μM, 27 for 1 μM, 23 for 10 μM and 22 for 100 μM. *p* < 0.001, one-way ANOVA followed by Dunnett's test. **(B)** Activation traces (left) and dose-response curve (right) of R-CaMP2 in neurons co-expressed with hK2α P212L in response to 30 photo-stimulations delivered at 20 Hz under 0, 1, 10, 100 μM Memantine. *n* = 22 for 0 μM, 27 for 1 μM, 23 for 10 μM and 22 for 100 μM. ***p < 0.001, one-way ANOVA followed by Dunnett's test. **(C)** Activation traces (left) and dose-response curve (right) of hK2α WT in response to 30 photo-stimulations delivered at 20 Hz under 0, 1, 10, 100 μM memantine. *n* = 22 for 0 μM, 22 for 1 μM, 23 for 10 μM and 23 for 100 μM. *p < 0.05, ***p < 0.001, one-way ANOVA followed by Dunnett's test. **(D)** Activation traces (left) and dose-response curve (right) of R-CaMP2 in neurons co-expressed with hK2α WT in response to 30 photo-stimulations delivered at 20 Hz under 0, 1, 10, 100 μM Memantine. *n* = 22 for 0 μM, 22 for 1 μM, 23 for 10 μM and 23 for 100 μM. ***p < 0.001, one-way ANOVA followed by Dunnett's test.

Our data suggest that pharmaceutical intervention on CaMKIIα activation is a reasonable candidate for ID caused by aberrantly facilitated Ca^2+^/CaM-dependent CaMKIIα activation. Since there is no approved CaMKII inhibitor for clinical use (Pellicena and Schulman, 2014; Nassal et al., 2020), we attempted to target upstream NMDAR-mediated Ca^2+^ influx using memantine, an NMDAR agonist clinically used for Alzheimer's disease (Parsons et al., 2007) and well-tolerated in children (Findling et al., 2007; Bouhadoun et al., 2021). Since memantine can potentially have pharmacological effects on targets other than NMDAR (Parsons et al., 2007; Moriguchi et al., 2018), we investigated whether memantine can potentially have an inhibitory effect on P212L in cultured neuron conditions. Multiplex imaging of R-CaMP2 and hK2α was performed in response to 30 photostimulations at 20 Hz in the presence of 0, 1, 10, and 100 μM of memantine. R-CaMP2 peak amplitude was decreased in a memantine concentration-dependent manner, with the signal being reduced to about half at 10 μM and most of the responses being lost at 100 μM (0 μM: 2.1 ± 0.056, *n* = 22; 1 μM: 1.9 ± 0.064, *p* = 0.56, *n* = 27; 10 μM 1.3 ± 0.17, *p* < 0.001, *n* = 23; 100 μM: 0.26 ± 0.068, *n* = 22, *p* < 0.001, compared to 0 μM, one-way ANOVA followed by Dunnett's test) (Figure 8B), suggesting inhibition of NMDAR. Accordingly, the responses of hK2α P212L were also attenuated at 10 and 100 μM memantine (0 μM: 0.32 ± 0.030, *n* = 22; 1 μM: 0.25 ± 0.032, *p* = 0.24, *n* = 27; 10 μM 0.13 ± 0.033, *p* < 0.001, *n* = 23; 100 μM: 0.020 ± 0.0054, *p* < 0.001, *n* = 22, compared to 0 μM, one-way ANOVA followed by Dunnett's test, compared with 0 μM) (Figure 8A). Similarly, WT hK2α showed a memantine concentration-dependent suppression of R-CaMP2 and hK2α responses (Figures 8C,D). These findings suggest that memantine may be a candidate agent for an interventional approach to suppress the accelerated Ca^2+^/CaM-dependent activation of P212L.

## Discussion

In this study, we identified the P212L *de novo* mutation in a patient with ID. Previous studies have examined the effect of P212L mutation on protein expression, threonine 286 phosphorylation, and cortical neuronal cell migration during development, but the effects of this mutation on the CaMKIIα at the molecular and cellular levels were not clarified. In this study, to examine Ca^2+^/CaM-dependent activation, which is fundamental to CaMKIIα function but had never been examined in P212L, we utilized our hK2α probe to develop a FRET-based optical molecular phenotyping system. Conventionally, Ca^2+^/CaM-dependent CaMKII activation has been performed by kinase assays measuring the incorporation of radiolabeled phosphate into substrates (De Koninck and Schulman, 1998). However, quantifying Ca^2+^/CaM-dependent activation using substrate-based readout can be complicated by different Ca^2+^/CaM-dependency between substrates (Coultrap et al., 2014) and kinase regulation mediated by Ca^2+^/CaM-dependent autophosphorylation of threonine 286, threonine 305, and threonine 306 (Cook et al., 2021), making it critical for direct readout of kinase activation state *per se*. Since hK2α probe reports activated conformation of the kinase (Fujii et al., 2013), it has the advantage of specifically detecting the activation state of the kinase without the need for the substrates, allowing for a more direct comparison of the mutations found in the kinase gene (Fujii and Bito, 2022). Thus, our FRET-based optical molecular phenotyping system provides a selective, sensitive, quantitative, and a scalable platform for the mutational analysis of the human CaMK2A gene. The platform will be applicable to other mutations in CAMK2A and CAMK2 isoforms related to various diseases (Iossifov et al., 2014; Küry et al., 2017; Akita et al., 2018; Chia et al., 2018; Brown et al., 2021; Proietti Onori and van Woerden, 2021; Mutoh et al., 2022) to reveal unappreciated molecular phenotypes in the future.

The present study clearly revealed that P212L mutation aberrantly facilitated Ca^2+^/CaM-dependent activation. P212L is located in the kinase domain at a hydrophobic core formed with the regulatory domain, and *in silico* analysis predicted that P212L substitution destabilized the hydrophobic core and impaired the interaction between the kinase and the regulatory domains (Akita et al., 2018). Adding our findings, it could be suggested that Ca^2+^/CaM can be more readily accessible or the regulatory domain can be more ready to release the kinase domain, which likely leads to a faster activation and a slower deactivation process in P212L.

High throughput FRET-based optical molecular phenotyping system revealed that aberrantly facilitated Ca^2+^/CaM-dependent activation was observed not only in P212L-specific molecular phenotype, but rather it was more widespread among CAMK2A *de novo* mutations associated with ID. So far, mutations associated with ID have been found in the kinase and the regulatory domains of CaMKIIα (Küry et al., 2017; Akita et al., 2018), while mutations found in schizophrenia patients were distributed in the kinase domain and the association domain (Brown et al., 2021). Since the kinase domain and the regulatory domain are involved in Ca^2+^/CaM-dependent activation, and the association domain is involved in dodecameric to tetradecameric holoenzyme formation, which is crucial for the regulation of autophosphorylation of threonine 286 through inter-subunit reaction, it raises the possibility that distinct molecular phenotypes of CaMKIIα can be underlying different disease phenotypes, and our data supports this hypothesis.

The mechanism of how the abnormal facilitation of Ca^2+^/CaM-dependent activation of P212L leads to the ID phenotype is currently unknown. However, in various animal models having CaMKIIα mutants with altered Ca^2+^/CaM-dependent activation, activation kinetics and frequency tuning of CaMKIIα have been shown to correlate with abnormalities in the regulation synaptic plasticity as well as learning and memory.

In CaMKIIα T286A knock-in mice, the frequency dependence of CaMKIIα activation and synaptic plasticity was tuned toward high-frequency input (Chang et al., 2017). The T286A knock-in mice had learning deficiency and required more repetition to form memory (Giese et al., 1998; Irvine et al., 2005). Transgenic mice constitutively expressing phosphor-mimicking mutant T286D had an altered frequency-tuning curve for synaptic plasticity that favored the induction of long-term depression at 5–10 Hz stimulation (Mayford et al., 1995). The mice showed impaired spatial memory (Bach et al., 1995) and abnormal properties of hippocampal place cells firing (Rotenberg et al., 1996), suggesting that abnormal plasticity tuning may induce altered network-level properties in the brain. Furthermore, in inducible T286D transgenic mice, in which transgene expression levels could be altered by changing Dox administration during development, high T286D expression suppressed hippocampal LTP, while low T286D expression promoted LTP (Mayford et al., 1996; Bejar et al., 2002). There were correlations between T286D expression and fear conditioning or water maize performance, consistent with our hypothesis that abnormal activation of CaMKIIα drives the behavioral phenotypes. In mutants in which the inhibitory phosphorylation sites of CaMKIIα, threonine 305, and threonine 306 were mutated with alanine, the dissociation of Ca^2+^/CaM was slower (Chang et al., 2019). In T305V/T306A mutant mice, although protein expression levels, abundant in the PSD, or threonine 286 autophosphorylation levels were comparable to the control, frequency tuning of long-term potentiation was tuned to lower frequency and flexibility in learning and the specificity of memory was reduced (Elgersma et al., 2002).

Based on these previous results and our results that P212L showed aberrantly enhanced Ca^2+^/CaM-dependent activation and frequency-response of CaMKIIα, we speculate that P212L mutation would lead to altered frequency tuning of synaptic plasticity tuning and induce deficiencies in learning and memory. However, facilitated Ca^2+^/CaM-dependent activation also possibly affects the level of Ca^2+^/CaM-stimulated phosphorylation of threonine 286, the level of inhibitory phosphorylation of threonine 305 and threonine 306, and binding to NMDAR. So, it is important to investigate these properties as well as to generate a knock-in mouse model of P212L in future studies.

Some of the mutants analyzed could have molecular phenotypes other than facilitated Ca^2+^/CaM-dependent activation. A112V mutant showed Ca^2+^/CaM-dependent activation similar to WT in a plate reader FRET assay but showed a significantly smaller response in living neurons. This suggests the possibility that molecular processes other than Ca^2+^/CaM-dependent activation could be disrupted in A112V, which remains to be elucidated. In E183V, although Ca^2+^/CaM-dependent modulation was significantly smaller compared to WT, consistent with the decreased catalytic activity of E183V (Stephenson et al., 2017), the baseline FRET ratio was unexpectedly elevated. A previous study had shown that CaMKIIα introduced with E183V mutation had enhanced ubiquitination and reduced stability (Stephenson et al., 2017). Therefore, it may be possible that accelerated degradation may break down the donor and acceptor of FRET probe, or may prohibit sufficient maturation of fluorescent proteins (Liu et al., 2018), increasing the baseline FRET ratio. P235L showed no significant changes in our optical molecular phenotyping system, although multiple comparison made phenotypic detection difficult. It is necessary to clarify the molecular phenotype of these mutations by combinatorially examining other molecular properties of CaMKIIα in future studies.

Our results suggest that WT is slightly more sensitive to memantine than P212L; however the underlying mechanism is currently not well-understood. Under conditions partially inhibited by memantine (1-10 μM), the influx of Ca^2+^ is partially reduced rather than completely blocked (Figure 8) and forms a lower concentration of Ca^2+^/CaM. Under these conditions, P212L, which can be activated at lower Ca^2+^/CaM concentrations (Figure 3), is likely to be activated more than WT. Furthermore, as the binding of activated CaMKIIα to GluN2B further enhances the interaction with Ca^2+^/CaM and results in an autonomous state (Strack and Colbran, 1998; Bayer et al., 2001), these molecular processes may possibly amplify CaMKIIα activation and lead to the differences in memantine sensitivity.

WT and P212L kinase subunits are considered to form hetero dodecamers in patients having heterozygous WT and P212L CAMK2A alleles, making it difficult to selectively inhibit P212L over WT pharmacologically. Since the dose-response curve of WT/P212L for memantine would be intermediate between the WT and the P212L, 1–10 μM of memantine could potentially reduce the aberrant activation of CaMKIIα in the WT/P212L to the same degree as in the WT under vehicle conditions. In future studies, the effectiveness of this approach needs to be assessed in knock-in model mice.

## Data availability statement

The whole-exome sequencing datasets presented in this study can be found in online repositories. The name of the repository and accession number can be found at: DNA Data Bank of Japan (DDBJ) Japanese Genotype-phenotype Archive (JGA), https://www.ddbj.nig.ac.jp/jga/index-e.html, JGAS000522. Other datasets that support the findings are available from the corresponding authors, HF and HB, upon reasonable request.

## Ethics statement

The studies involving human participants were reviewed and approved by the Ethics Committee of the Nagoya University Graduate School of Medicine. Written informed consent to participate in this study was provided by the participants' legal guardian/next of kin. The animal study was reviewed and approved by the institutional review committees of the University of Tokyo Graduate School of Medicine.

## Author contributions

HF, HK, ST-K, and HB conceived the study. HF performed plasmid construction, multiplex imaging, plate reader assays, statistical analysis, and wrote the manuscript. HK collected clinical data, assisted in data interpretation and manuscript preparation, and reviewed the manuscript. YK performed plasmid construction and plate reader assays. MK collected clinical data, assisted in data interpretation, and reviewed the manuscript. SH performed Sanger sequence. JN coordinated and supervised data collection and critically reviewed the manuscript for important intellectual content. All authors contributed to the article and approved the submitted version.

## Funding

This work was supported in part by grants from Grant-in-Aid for Brain Mapping by Integrated Neurotechnologies for Disease Studies (Brain/MINDS) (JP19dm0207079 to HB), Brain Information Dynamics (BID) (JP17H06312 to HB), JSPSKAKENHI (JP17K13270 to HF, JP22H00432, and JP22H05160 to HB, and JP21H05091 to ST-K), Takeda Science Foundation (to HF and HB), Nakatani Foundation (to HB), Astellas Foundation for Research on Metabolic Disorders (to HF), Hitachi Global Foundation (to HB), and the Toray Science Foundation (ST-K).

## Conflict of interest

The authors declare that the research was conducted in the absence of any commercial or financial relationships that could be construed as a potential conflict of interest.

## Publisher's note

All claims expressed in this article are solely those of the authors and do not necessarily represent those of their affiliated organizations, or those of the publisher, the editors and the reviewers. Any product that may be evaluated in this article, or claim that may be made by its manufacturer, is not guaranteed or endorsed by the publisher.

## Supplementary material

Supplementary Figure 1 R-CaMP2 Responses in Living Neurons co-Expressing hK2α Mutants Associated With ID. **(A–J)** R-CaMP2 activation kinetics in response to 30 photo-stimulations delivered at 5 Hz (left) and 20 Hz (right) To aid comparison, response curve of R-CaMP2 co-expressed with hK2α WT are overlayed (shaded blue traces) in each mutant data [red traces, **(B–J)**]. Mean ± s.e.m. are shown. *n* = 14 for E183V and H282R, *n* = 15 for WT, E109D, and T286P, *n* = 16 for F98S, A112V, P212L, P212Q, and P235L.

Supplementary Figure 2 Comparison of hK2α and R-CaMP2 responses. **(A–D)** Peak amplitude of hK2α and R-CaMP2 responses in response to photo-stimulations delivered at 5 Hz **(A,B)** or 20 Hz **(C,D)**. Each dot plots represent data from each neuron and black bar represents mean. ^*^*p* < 0.05, ^**^*p* < 0.01, ^***^*p* < 0.001, one-way ANOVA followed by Dunnett's test compared with WT. *n* = 14 for E183V and H282R, *n* = 15 for WT, E109D, and T286P, *n* = 16 for F98S, A112V, P212L, P212Q, and P235L.
