# Supplementary figures and images for: Förster resonance energy transfer-based kinase mutation phenotyping reveals an aberrant facilitation of Ca2+/calmodulin-dependent CaMKIIα activity in de novo mutations related to intellectual disability
Source: Front Mol Neurosci. 2022 Sep 1;15:970031. doi: 10.3389/fnmol.2022.970031 (PMC9474683; doi:10.3389/fnmol.2022.970031)

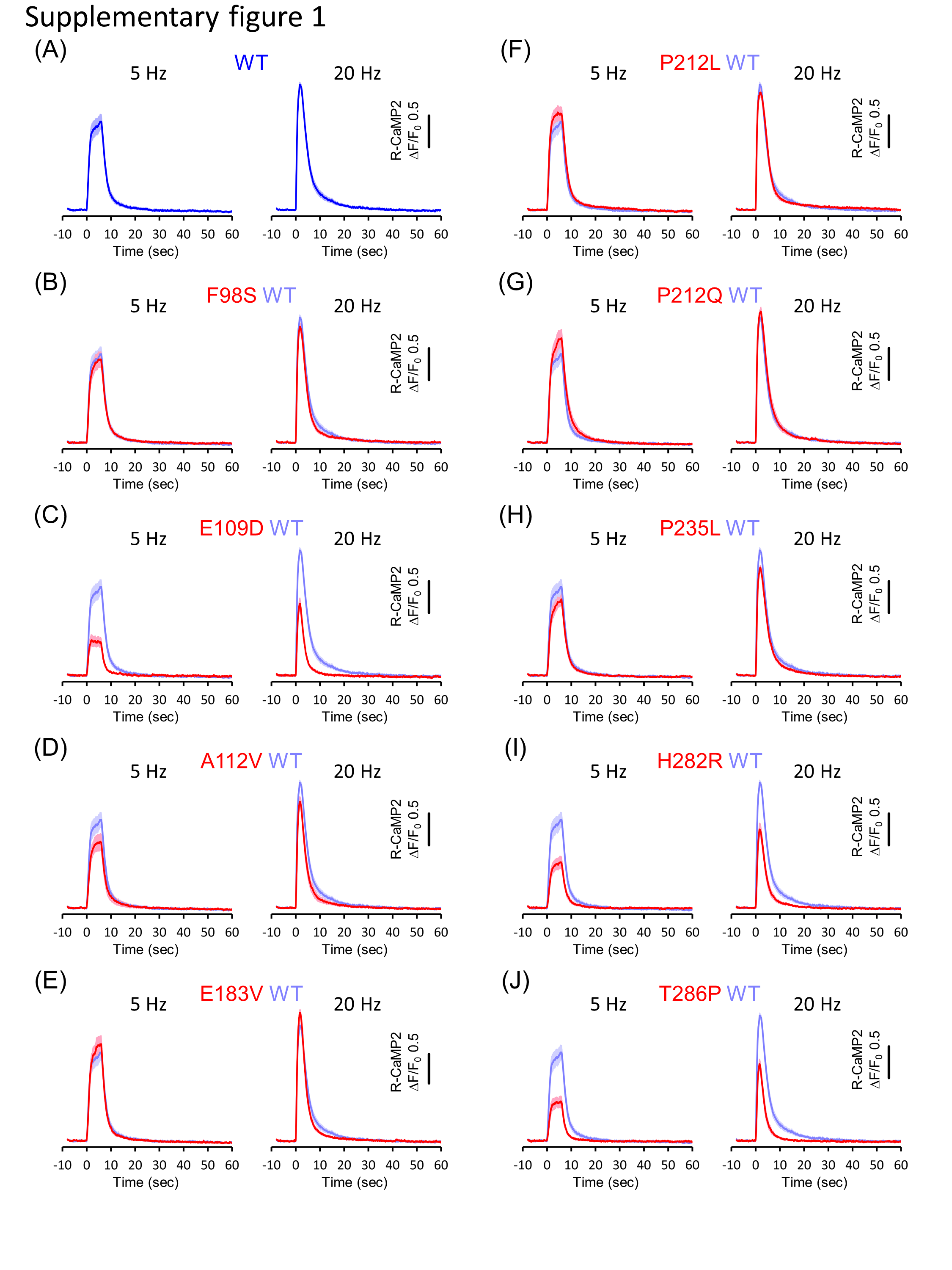

Supplement: Supplementary Figure 1 — R-CaMP2 Responses in Living Neurons co-Expressing hK2α Mutants Associated With ID. (A–J) R-CaMP2 activation kinetics in response to 30 photo-stimulations delivered at 5 Hz (left) and 20 Hz (right) To aid comparison, response curve of R-CaMP2 co-expressed with hK2α WT are overlayed (shaded blue traces) in each mutant data [red traces, (B–J)]. Mean ± s.e.m. are shown. n = 14 for E183V and H282R, n = 15 for WT, E109D, and T286P, n = 16 for F98S, A112V, P212L, P212Q, and P235L. [file Image_1.tif]

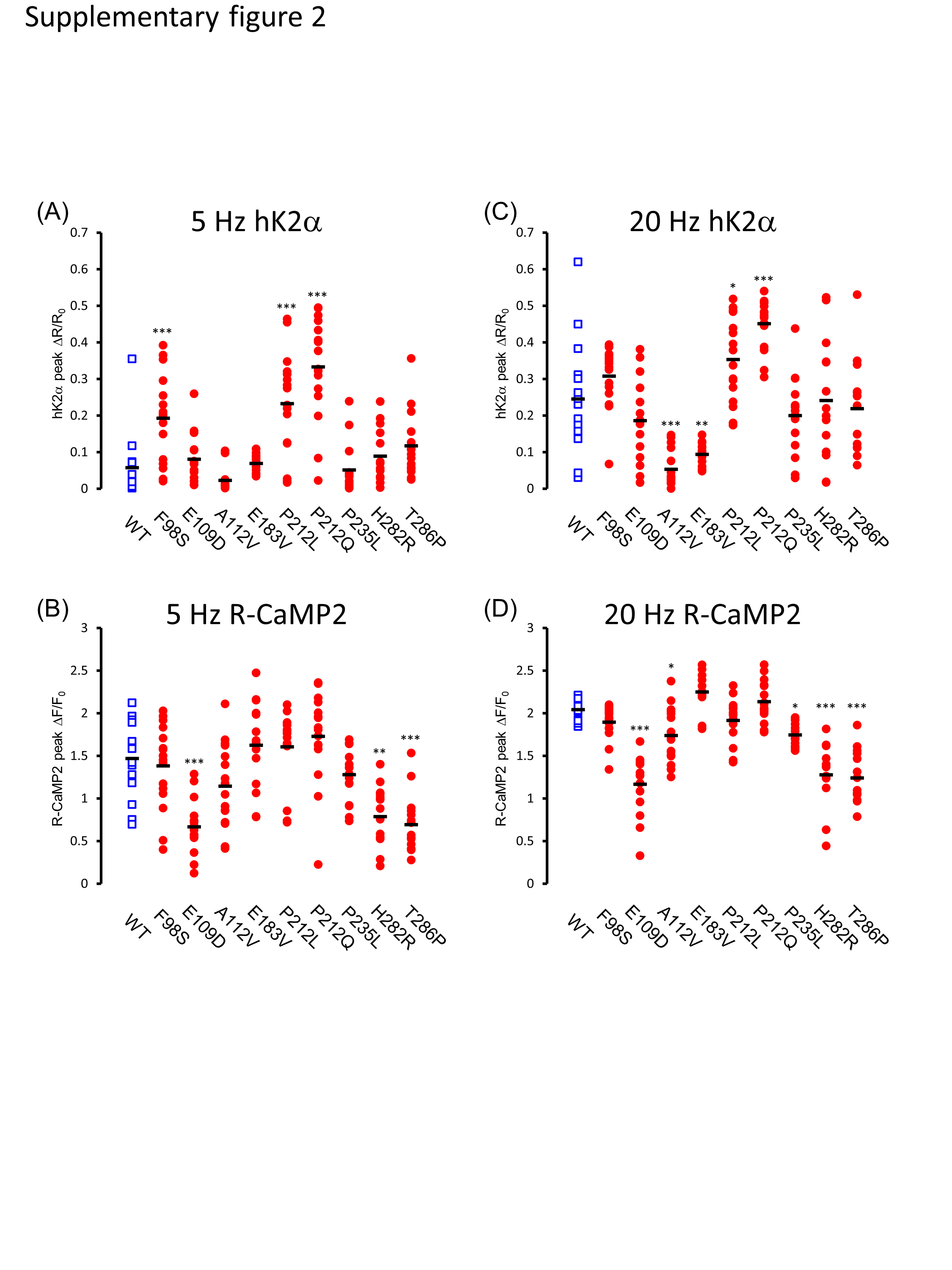

Supplement: Supplementary Figure 2 — Comparison of hK2α and R-CaMP2 responses. (A–D) Peak amplitude of hK2α and R-CaMP2 responses in response to photo-stimulations delivered at 5 Hz (A,B) or 20 Hz (C,D). Each dot plots represent data from each neuron and black bar represents mean. *p < 0.05, **p < 0.01, ***p < 0.001, one-way ANOVA followed by Dunnett's test compared with WT. n = 14 for E183V and H282R, n = 15 for WT, E109D, and T286P, n = 16 for F98S, A112V, P212L, P212Q, and P235L. [file Image_2.tif]
